# Exploring an Ecologically Sustainable Scheme for Landscape Restoration of Abandoned Mine Land: Scenario-Based Simulation Integrated Linear Programming and CLUE-S Model

**DOI:** 10.3390/ijerph13040354

**Published:** 2016-03-24

**Authors:** Liping Zhang, Shiwen Zhang, Yajie Huang, Meng Cao, Yuanfang Huang, Hongyan Zhang

**Affiliations:** 1 College of Resources and Environmental Sciences, China Agricultural University, Beijing 100193, China; zhangliping0922@163.com (L.Z.); yajiehuang_cau@163.com (Y.H.); ciymeng@163.com (M.C.); 2 College of Earth and Environmental Sciences, Anhui University of Science and Technology, Huainan 232001, China; mamin1190@126.com; 3 College of Science, China Agricultural University, Beijing 100193, China; hongyan@cau.edu.cn

**Keywords:** land conservation, abandoned mine land transformation, scenario simulation, linear programming model, CLUE-S model, ecological restoration

## Abstract

Understanding abandoned mine land (AML) changes during land reclamation is crucial for reusing damaged land resources and formulating sound ecological restoration policies. This study combines the linear programming (LP) model and the CLUE-S model to simulate land-use dynamics in the Mentougou District (Beijing, China) from 2007 to 2020 under three reclamation scenarios, that is, the planning scenario based on the general land-use plan in study area (scenario 1), maximal comprehensive benefits (scenario 2), and maximal ecosystem service value (scenario 3). Nine landscape-scale graph metrics were then selected to describe the landscape characteristics. The results show that the coupled model presented can simulate the dynamics of AML effectively and the spatially explicit transformations of AML were different. New cultivated land dominates in scenario 1, while construction land and forest land account for major percentages in scenarios 2 and 3, respectively. Scenario 3 has an advantage in most of the selected indices as the patches combined most closely. To conclude, reclaiming AML by transformation into more forest can reduce the variability and maintain the stability of the landscape ecological system in study area. These findings contribute to better mapping AML dynamics and providing policy support for the management of AML.

## 1. Introduction

The intensive extraction of mining resources has pushed a considerable number of mines toward resource exhaustion, leading to large abandoned mine land (AML) areas. AML refers to land that has been disturbed or contaminated by mining or exploration activities and thus cannot be utilized without some type of remediation [1]. AML not only occupies large valuable land resources, but also leads to some detrimental effects, such as high levels of soil pollution, soil erosion, landslides, and land desertification [1,2,3,4]. Resource-exhausted cities are in urgent need of industrial transformation to realize sustainable development, which means that large amounts of construction land are required to build new factories, while cultivated land is also needed to support the increasing population. Moreover, most AML sites are in the center or on the edges of cities [1], which can severely affect urban development and public safety during rapid urbanization. In China, land consolidation (land reclamation included [1,5]) is an indispensable way of spatial reconstruction, which is praised as a means to share the benefits of providing new arable land and strengthening the utilization of already available land resources [6,7]. The government has committed huge investments to convert AML into other land-use types such as cultivated land, construction land, and forest land in order to adjust land-use structure and protect regional ecological safety. Therefore, scientifically simulating the change of AML and then exploring the ecological sustainable scheme have become necessary for sustainable development and ecological restoration in mining cities.

However, current research on AML mostly focuses on soil and vegetation rehabilitation at the micro level. For example, Bendfeldt *et al.* [8] studied the long-term effects (*i.e.*, over 16 years) of organic amendments on soil quality of amended mine soils in Virginia (United States), while Alday *et al.* [9] examined how soil and environmental factors influence the vegetation succession on reclaimed coal wastes in Spain. Previous scholars have also demonstrated some interest in legislation, policy, and management at the macro level. Soltanmohammadi *et al.* [10] offered an analytical approach for post-mining land-use determination in Iran. Sullivan *et al.* [11] proposed hardwood tree planting and a forest reclamation policy for improving reclaimed surface mine land in the Appalachian coal region of the United States. Mishra *et al.* [12] analyzed the costs of abandoned coal mine reclamation in Ohio (United States) and provided evidence of potential Pareto improvement by investing limited resources in reclamation projects. Hu *et al.* [13] revealed concurrent mining and land reclamation plans can enhance the quality of cultivated land and provide better land protection and food security in mined areas in China. However, limited information is available on how to conduct the spatial reconstruction of AML and on which reclamation scenario is more suitable for ecological restoration in mining cities.

The core objective of AML reclamation is to convert AML into other land-use types. Land/cover change models are an important tool to understand the driving forces and processes of land-use changes, assess the ecological impact of land-use changes, and make decisions regarding land-use planning [14,15,16,17,18,19,20,21,22,23,24,25,26,27]. Several models have been used to map land-use changes, such as the Agent-based model, Cellular Automata model, System Dynamic model, and the Conversion of Land Use and its Effects at Small Regional Extent (CLUE-S) model.

The CLUE-S model was developed to simulate land use change using empirically quantified relations between land use and its driving factors in combination with dynamic modeling of competition between land use types [28,29,30,31,32,33,34,35,36,37,38,39,40,41]. This model has been recognized as an excellent tool to simulate land-use changes. However, although the CLUE-S model is preferred to address the spatial allocation of land-use change, it also has some limitations because it requires another mathematical model to calculate future land-use demand.

Based on the foregoing, this study adopts the linear programming (LP) model to take full consideration of AML dynamics and understand future land requirements for all land-use types. The CLUE-S model is used to map the spatial transformations of AML for 2020 in the Mentougou District (Beijing, China). Three scenarios are created to map the spatial distribution of land-use types by using 2007 as a baseline: the planning scenario based on the general land-use plan in Mentougou District (scenario 1), maximal comprehensive benefits (scenario 2), and maximal ecosystem service value (ESV) (scenario 3). Next, landscape pattern changes are analyzed by using several landscape-scale metrics. Briefly, this study addresses the following objectives: (i) how to design multi-scenario characteristics for simulating AML transformations using the coupled LP and CLUE-S models and (ii) how to analyze landscape pattern changes based on simulated maps and determine which scenario is better for ecological sustainable development.

## 2. Materials and Methods

### 2.1. Study Area

**Figure 1:**
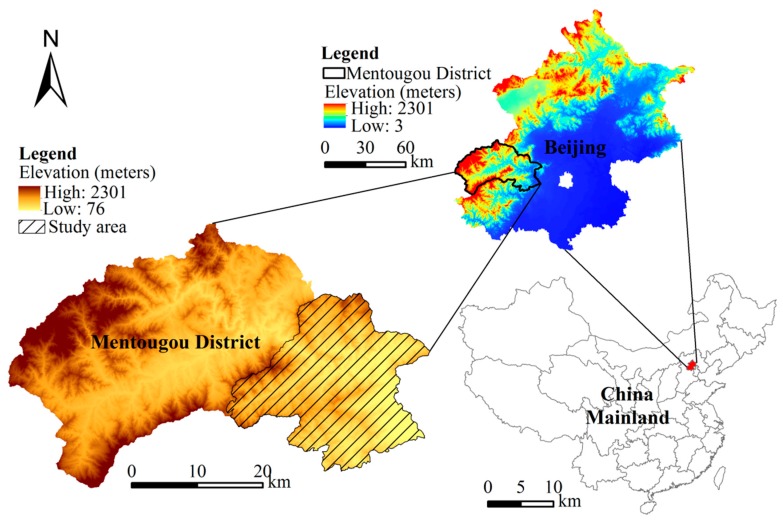
Site and elevation map for the study area.

Mentougou District (39°48′–40°10″ N, 115°25′–116°10″ E) is located in the western region of Beijing, China, where mining resources are rich and the main minerals are coal and limestone. It was once the energy base of Beijing; however, in 2007, the Beijing Municipal Government shifted its attention to mining in the district, where ecological preservation was later proposed. Thus, the local government began shutting down mines for land reclamation. From 2002 to 2008, 267 mines were closed, resulting in 4130 hm^2^ of AML. According to the *General Land-Use Plan in Mentougou District (2006–2020)*, reclaiming and utilizing AML is an effective way of improving the ecological environment and adjusting land-use structures in mining cities. Seven towns in Mentougou District were selected as research areas: Junzhuang, Yongding, Miaofengshan, Wangping, Longquan, Tanzhesi, and Datai. However, the spatial allocation of Yongding is not continuous: while one part is located in the middle of the study area, the other is in the southeast. Similarly, for Wangping, two parts of the town are located in the east and west of the Datai, respectively. The site and elevation map of the study area are shown in Figure 1.

### 2.2. Data and Driving Factors

#### 2.2.1. Land-Use Categories Classification

Based on land use properties in study area and *Current Land Use Classification in China* (GB/T 21010–2007), the land-use map was classified into eight first land-use categories, including cultivated land, garden land, forest land, grassland, construction land, AML, water, and unutilized land.

**Figure 2:**
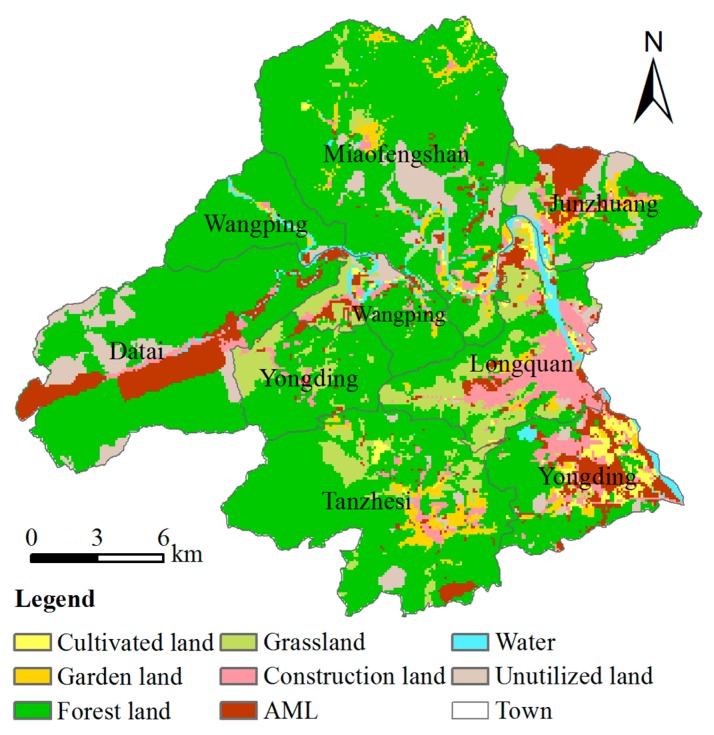
Land-use map of the study area (2007).

Data for the spatial distribution of land-use types in the base year were taken from the 2007 land-use map of Beijing from Bureau of Land Resources, Beijing. The respective areas for eight land-use types are 831 hm^2^, 1678 hm^2^, 30165 hm^2^, 3686 hm^2^, 2733 hm^2^, 3573 hm^2^, 905 hm^2^, and 3063 hm^2^ (Figure 2).

The land-use information for 2013, which was extracted from Landsat TM image data (acquired in 2013; resolution: 30 m) and processed by human-computer interactive operations, was used to evaluate the accuracy of the CLUE-S model’s simulation.

#### 2.2.2. Choices of Driving Factors

CLUE-S model is driven by geophysical and socioeconomic variables. In this study, 15 driving factors were included on the basis of their availability, stability, relevance, and data suitability: elevation (*X*_1_), slope (*X*_2_), distance to the nearest road (*X*_3_), distance to the nearest railroad (*X*_4_), distance to the nearest river (*X*_5_), distance to the nearest main town (*X*_6_), distance to the nearest rural resident site (*X*_7_), soil organic matter (*X*_8_), population density (*X*_9_), per capita income (*X*_10_), agricultural population (*X*_11_), mining industry practitioners (*X*_12_), crop yield (*X*_13_), annual rainfall (*X*_14_), and annual afforestation areas (*X*_15_).

All the driving factors (*X*_1_–*X*_15_) were selected on the basis of expert knowledge, with taking the availability, stability, and suitability of data into consideration. Experts marking method was used to determine the driving variables. We consulted ten experts whose research interests focus on mined land reclamation and they selected 20 driving factors at first. Afterwards, we examined the correlation of the selected factors and removed the highly relevant factors through redundancy analysis, leaving 15 factors finally.

**Figure 3:**
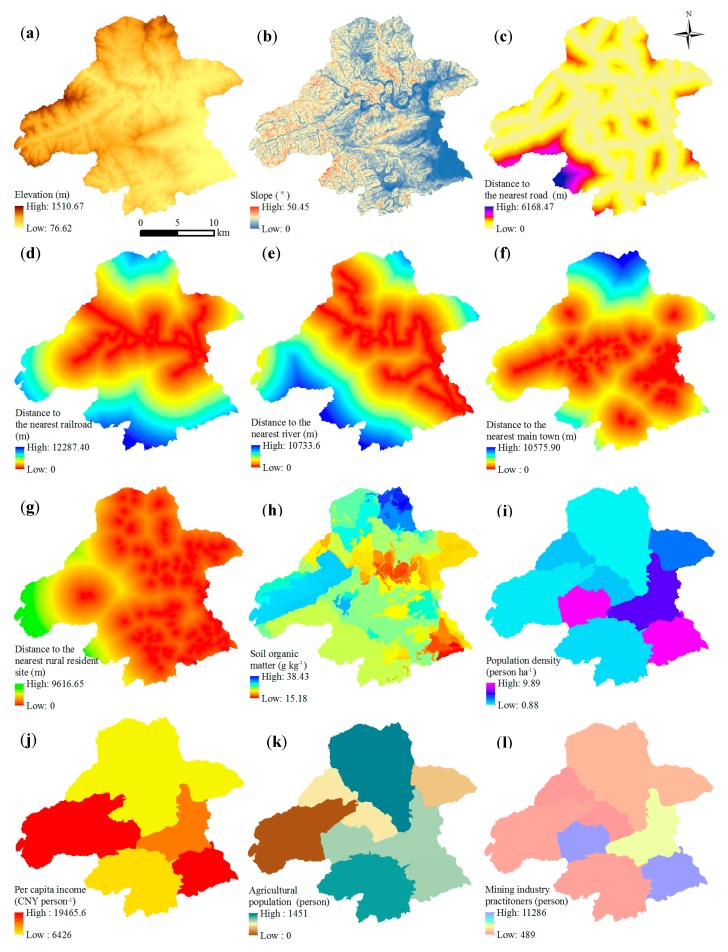

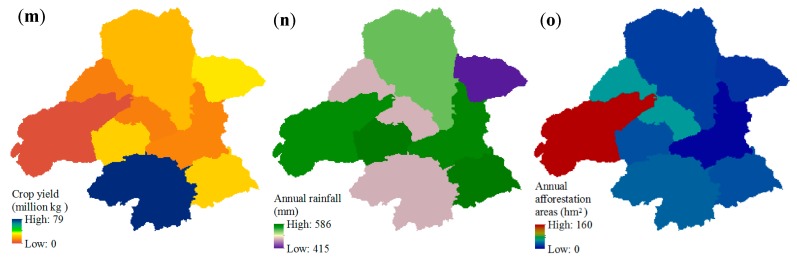
Maps of the driving forces in the study region. (**a**) Elevation (m); (**b**) Slope (˚); (**c**) Distance to the nearest road (m); (**d**) Distance to the nearest railroad (m); (**e**) Distance to the nearest river (m); (**f**) Distance to the nearest main town (m); (**g**) Distance to the nearest rural resident site (m); (**h**) Soil organic matter (g kg^−1^); (**i**) Population density (person ha^−1^); (**j**) Per capita income (CNY person^−1^); (**k**) Agricultural population (person); (**I**) Mining industry practitioners (person); (**m**) Crop yield (million kg); (**n**) Annual rainfall (mm); (**o**) Annual afforestation areas (hm^2^).

Specifically, *X*_1_ and *X*_2_ were used to describe the terrain conditions in the study area. Higher evaluation and slope can restrict the transformation of AML into cultivated land. *X*_3_, *X*_4_, *X*_5_, *X*_6_, and *X*_7_ were used to describe transportation accessibility. A good transport condition can be suitable for AML shifted into construction land. *X*_8_ was one of the most important indicators of soil properties, which is essential for the reclamation of AML for cultivation. *X*_9_, *X*_10_, *X*_11_, X_12_, and X_13_ were the key socioeconomic factors influencing the transformation of all land-use categories. *X*_14_ and *X*_15_ were the indicators reflecting climatic conditions and the policy-oriented behaviours of ecological protection, respectively. Figure 3 shows the maps of the driving forces in the study region.

As for the geophysical variables, elevation and slope data were acquired by using a digital evaluation model (DEM). Besides, soil organic matter was derived from a 1:5 million soil map, and the distance to the nearest road, railroad, river, main town, and rural resident sites were calculated by using ArcGIS 10.0 to describe transport accessibility. What is more, the DEM; soil map; and distribution of road, railroad, river, main town, and rural resident sites were obtained from *Beijing Digital Soil System*.

Socioeconomic variables for 2007 such as population density, per capita income, agricultural population, mining industry practitioners, crop yield, annual rainfall, and afforestation area were obtained from the *Statistical Yearbooks of the Mentougou District, Beijing*.

The CLUE-S model is a grid-based model, and the spatial scale refers to spatial resolution (*i.e.*, the raster cell size). In this study, all GIS data were converted into an equal-area projection and gridded by using a basic grid size of 100 × 100 m. The same approach was followed for the socioeconomic variables.

### 2.3. Methods

Generally, in this study, we developed LP and CLUE-S models to characterize the land-use change process after land reclamation. The scenario simulation of AML conversions markedly differed from general land-use changes, given that the types of AML significantly changed before and after reclamation. The LP model depicted the direction of land-use shifts and calculated future land-use requirements for land-use categories by taking account of the Mined Land Suitability (see Section 2.3.1) constraints and land-use planning restrictions. All land-use changes should identify with the general land-use plan issued by the local government. For example, the areas of certain land-use types such as cultivated land and forest land should not be lower than the specified numbers in the land-use plan during the process of urbanization to maintain the ecological balance in urban cities. The specific numbers above were considered constraints of the LP model. All the demand for land-use categories can be calculated via LP model. So given these features, LP model was suitable for predicting future demand in the study area with a large amount of AML needed to be reclaimed to other land-use types. The CLUE-S model can allocate the land requirements to spatial allocation driven by the driving forces. The details about the method can be found below.

#### 2.3.1. Mined Land Suitability (MLS) Assessment

The suitability of mined land is a critical factor for AML conversion. According to the natural and socioeconomic conditions of the Mentougou District, Beijing, AML has potential to be reclaimed into cultivated land, garden land, forest, and construction land. Hence, a limit condition method was employed to assess the suitability of mined land. Each AML patch was treated as one assessment unit (in total 93 patches were included).

(1) Indexes system and evaluation criterion

Seven factors were selected to assess mined land suitability. The main limiting factors of land reclamation and evaluation criteria of cultivated land, garden land, forest, and construction land are shown in Table 1.

Based on the investigation of land quality, the actual score of each assessment factor of the assessment unit can be compared with the evaluation criteria in Table 1. The two-level evaluation system was divided into suitability categories and suitability classes. Suitability categories contained suitable as well as unsuitable categories (*i.e.*, N in Table 1), while suitability classes were subdivided into the first, second, and third classes (*i.e.*, 1, 2, 3 in Table 1).

(2) Confirmation of suitable land-use types for AML

The limit condition method, which is based on the “cask principle”, emphasizes the function of dominant limiting factors:

(1) $$Y_{i}=\min(Y_{ij})$$

where *Y_i_* is the score of the evaluation unit of *i* and *Y_ij_* is the score of the evaluation factor of *j* in the evaluation unit of *i*.

Finally, we should account for policy, ecological, and economic factors as well as public participation to finalize the results. The MLS assessment result can be used as a constraint in the LP model.

#### 2.3.2. LP Model

The CLUE-S model was divided into two modules: the non-spatial module and the spatial module. We used the LP model to calculate the demand for all land use in the non-spatial module, while the spatial module translates this demand into land-use changes according to the probabilities and rules of different land-use types in the CLUE-S model.

Three land-use maps were simulated for 2020, assuming three potential modes of development on the basis of land reclamation rules, and policy-oriented behaviors. Demand for all land-use types in scenario 1 was obtained from the *General Land-Use Plan for Mentougou District (2006–2020)* and that for scenarios 2 and 3 was derived from the results of the LP model on the basis of the objective function of the niche method of land use [42] and ESV method [43], respectively.

##### Scenario 1

The restrictions on land use in this scenario conform to *General Land-Use Plan for Mentougou District (2006–2020)*. This plan stated that during reclamation and urbanization development, the cultivated land, garden land, forest land, and construction land in the study area for 2020 should be kept at least 1656 hm^2^, 1678 hm^2^, 29,738 hm^2^, and 6688 hm^2^, respectively, to balance the local land-use structure. Demand for the other land-use types was also derived from the general land-use plan.

##### Scenario 2

The LP model based on the niche method of land use was applied to optimize the reutilization of AML. A land-use type niche refers to the structural relationship among different land-use types, area ratio, utilization efficiency, and the mutual transformation between different types. It includes three parts: a natural niche (*i.e.*, land quality, utilization intensity, and food security), an economic niche (economic benefits), and a social niche (*i.e.*, policies and regulations). The total niche of land use is the capacity of a land-use type to take over a new environment. Theoretically, land-use changes are subject to the total niche of land use. The total land-use niche is calculated as follows:

(2) $$TN_{i}=NN_{i}\times 0.4+EN_{i}\times 0.4+SN_{i}\times 0.2$$

where *TN_i_* is the total niche of a certain type of land use. *NN_i_*, *EN_i_*, and *SN_i_* are the natural, economic, and social niches, respectively. The weights of *NN_i_*, *EN_i_*, and *SN_i_* are, respectively, 0.4, 0.4, and 0.2 [42]. The *i* values range from 1 to 8 for the eight land-use categories. The parameters are shown in Table 2. 

The objective function for scenario 2 can be seen in Equation (3):

(3) $$Z=\max\left(\begin{array}{l} TN_{1}\times (x_{1}+x_{9})+TN_{2}\times (x_{2}+x_{10})+TN_{3}\times (x_{3}+x_{11})+TN_{4}\times x_{4}\\ +TN_{5}\times (x_{5}+x_{12})+TN_{6}\times x_{6}+TN_{7}\times x_{7}+TN_{8}\times x_{8} \end{array}\right)$$

where *Z* is the objective function for scenario 2. *x*_1_, *x*_2_, *x*_3_, *x*_4_, *x*_5_, *x*_6_, *x*_7_, and *x*_8_ represent the eight land-use types. *x*_9_, *x*_10_, *x*_11_, and *x*_12_ represent the areas of AML reclaimed to cultivated land, garden land, forest land, and construction land, respectively. All *x_s_* can be calculated by using the constraints of the LP model as follows:

(4) $$\left\{ \begin{array}{l} x_{1}+x_{2}+x_{3}+...+x_{11}=46634\\ x_{9}+x_{10}+x_{11}+x_{12}=3072\\ x_{6}=501\\ x_{9}\leq 85.3\\ x_{10}\leq 176.1\\ x_{11}\leq 1729.0\\ x_{12}\leq 1081.5\\ x_{1}+x_{9}\geq 1656\\ x_{2}+x_{10}\geq 1678\\ x_{3}+x_{11}\geq 29738\\ x_{5}+x_{12}\geq 6688 \end{array}\right.$$

where the first three inequations represent the total area of the study region, total reclamation area of AML, and area of AML in 2020 without reclamation, respectively. The following four inequations are the results for MLS and *x*_9_, *x*_10_, *x*_11_, and *x*_12_ are the respective areas of AML suitable for reclamation to cultivated land, garden land, forest land, and construction land. The last four inequations denote the minimal area of cultivated land, garden land, forest land, and construction land in the study area for 2020 according to the *General Land-Use Plan in Mentougou District (2006–2020)*. 

##### Scenario 3

ESV method was used to evaluate the ecological benefits of the simulated maps. Ecosystem services comprise the flows of material, energy, and information from natural capital stocks, which combine with manufactured and human capital services to produce human welfare [43]. The ESVs for the eight land-use types are shown in Table 3.

AML can be reclaimed as cultivated, garden, forest and construction land on the basis of the natural and social conditions in the study area. However, the ESV of construction land (0 CNY·hm^−2^) is much lower than that of forest land (11,735.57 CNY·hm^−2^) (Table 3). Therefore, it is suitable for reclamation to forest land to meet the demand of maximal ESV. Equation (5) is the objective function for this scenario and the constraints are the same as in scenario 2 (but with *x*_12_ = 0). All the *x*_s_ can be calculated by using the constraint and the objective function can be seen as follows:

(5) $$Z’=\max\left(\begin{array}{l} ESV_{1}\times (x_{1}+x_{9})+ESV_{2}\times (x_{2}+x_{10})+ESV_{3}\times (x_{3}+x_{11})+ESV_{4}\times x_{4}\\ +ESV_{5}\times x_{5}+ESV_{6}\times x_{6}+ESV_{7}\times x_{7}+ESV_{8}\times x_{8} \end{array}\right)$$

where *Z’* is the objective function for Scenario 3. *ESV*_1_, *ESV*_2_, *ESV*_3_, *ESV*_4_, *ESV*_5_, *ESV*_6_, *ESV*_7_, and *ESV*_8_ represent the ESVs of the eight land-use categories. Therefore, demand for all land-use types under the three scenarios for 2020 can be calculated as above. Further, the land requirements for these land-use types between 2007 and 2020 can be obtained by using the linear interpolation method.

#### 2.3.3. CLUE-S Model

The spatial module in the CLUE-S model comprises a spatially explicit allocation procedure. The spatial distributions of the land-use types are quantified by using a binomial logit model with the percentages of the types as the dependent variables and geomorphologic, transportation access, soil-related, and socioeconomic driving factors as the independent variables.

In this study, 15 driving factors were included as showed in Section 2.2.2. The probabilities of the conversion of location characteristics were defined by using the following logit model:

(6) $$\ln(\frac{P_{i}}{1-P_{i}})=\beta_{0}+\beta_{1}X_{1,}{}_{i}+\beta_{2}X_{2,i}+...+\beta_{n}X_{n,i}$$

where *P_i_* is the probability that a grid cell in location *i* contains a particular type and the *X_i_* represent the driving factors . The coefficient (*β*) is estimated by using a logit regression with the actual type as the dependent variable and *i* values ranging from 1 to 8 for the eight land-use categories. The *n* values range between 1 and 15 for *X*_1_–*X*_15_, respectively. The conversion between the different types determines the changes that will eventually take place. The specific conversion settings of the eight types affect the temporal dynamics of the simulation, which are composed of two parameters: conversion elasticity (*ELAS*) and the transition matrix. The first parameter, which ranges from 0 (easy conversion) to 1 (irreversible change), is determined on the basis of expert knowledge and observed behavior in recent years. The value for the second parameter, the transition matrix, is 0 (irreversible transition) or 1 (easy conversion) and indicates the possible conversions for each type. In this study, the other seven land-use types were not allowed to be converted into AML. In addition, construction land and water were not allowed to be converted into other land-use types because of their stable characteristics. The CLUE-S model operates in discrete time steps and uses conversion rules to simulate demand for all patterns and the most likely changes in the different types on the basis of Equation (6). For each grid *i*, the total probability (*TPROP_i,u_*) was calculated for each type according to the following formula:

(7) $$TPROP_{i,u}=P_{i,u}+ELAS_{u}+ITER_{u}$$

where *TPROP_i,u_* is the suitability of location *i* for type *u*, *ELAS_u_* is the conversion elasticity for type *u*, and *ITER_u_* is an iteration variable specific to type *u* and indicative of the relative competitive strength of type *u*.

#### 2.3.4. Landscape-Scale Graph Metrics Selection

Landscapes contain complex spatial patterns in the distribution of resource that vary over time. Landscape pattern analysis quantifies these patterns and their dynamics. The landscape patterns of simulated maps tend to differ by patch area and by the spatial location of patch types under various reclamation scenarios. It is widely known that landscape indices can describe information on landscape patterns, reflect the characteristics of structural compositions and spatial configurations, and quantitatively describe and monitor landscape structural changes over time. In this study, we used nine landscape-level graph metrics to reflect the range of the landscape characteristics of the simulated maps according to their ecological meanings (Table 4) in order to determine the optimal scenario.

## 3. Results

### 3.1. Quantitative Analysis of Land-Use Categories

The demand for all land-use categories calculated by LP model under the three scenarios for 2020 is shown in Table 5. The interplay among the land requirements, land-use policy, and competition for the eight land-use types resulted in differences in land-use dynamics among these scenarios. 

The land-use conversions were simulated by CLUE-S model from 2007 to 2020. To reveal the trend of land-use changes, we compared the simulated results with the demand to analyze the dynamics of land-use types (Table 5). We can see that the relative error between them were small.

### 3.2. Regression Analysis of Land-Use Changes

A logistic regression model was used to explore the relationship between land-use changes and the related driving forces. The logistic regression results were further examined by using *Receiver Operating Characteristic* (*ROC*) curves. An *ROC* greater than 0.7 suggests strong correlations and abilities to explain the conversion between the different types using the selected driving forces. *ROCs* for eight land-use types were 0.910, 0.874, 0.789, 0.818, 0.906, 0.847, 0.946, and 0.790, respectively. They were all above 0.7, which revealed that the spatial distribution for all land-use types can be explained by the selected driving forces, whereas different driving forces result in some differences in various land-use types. The resulting regression coefficients for the driving forces were used in the subsequent experiments.

### 3.3. Spatial Distribution of Simulated Maps

**Figure 4:**
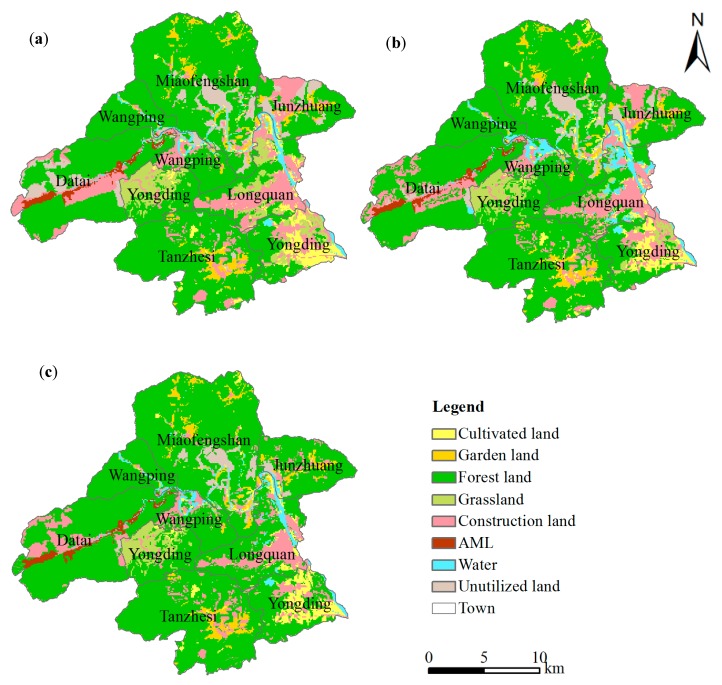
Simulated maps under the three scenarios for 2020: (**a**) scenario 1; (**b**) scenario 2; and (**c**) scenario 3.

The spatial distributions all land-use types for the three scenarios in 2020 are presented in Figure 4. Under scenario 1, cultivated land was mainly concentrated in Yongding with an area of 839 hm^2^ (Figure 4a). The garden land area was more or less the same as that in 2007, while its spatial distribution differed. Garden land in Yongding declined, whereas that in Miaofengshan increased. Forest land declined with an area of 360 hm^2^ and grassland showed an obvious tendency of decline with an area of 1027 hm^2^. Most grassland was converted into forest land. Water increased by 45 hm^2^. According to the land-use plan, there is a great need for construction land to foster industrial transformation and urbanization. Most AML was reclaimed to construction land to meet the demands of that in the non-spatial module. The reduced unutilized land was shifted into construction land as well. The situation conformed to the demand for supplying cultivated land and construction land represented in the local land-use planning.

Under scenario 2, there was a rising tendency in the amount of cultivated land, forest land, water, and construction land, which increased by 844 hm^2^, 996 hm^2^, 735 hm^2^, and 3928 hm^2^, respectively (Figure 4b). However, there was a sharp decline in grassland and unutilized land, with declining areas of 1984 hm^2^ and 1420 hm^2^. Garden land was more or less the same.

Under scenario 3, there was an increasing tendency in the amount of cultivated land, forest land, water, and construction land, which increased by 838 hm^2^, 2637 hm^2^, 175 hm^2^, and 3947 hm^2^, respectively (Figure 4c). However, there was a sharp decline in grassland and unutilized land, with declining areas of 2588 hm^2^ and 1956 hm^2^. Again, garden land was more or less the same.

Cultivated land was mainly seen in Yongding, which is located in the southeast region of the study area (Figure 4). As shown in Equation (7), *TPROP* was assumed to provide an accurate description of the spatial distribution of all types under the actual geophysical and socioeconomic conditions in the study area. The conversion was observed in the grid cells with higher *TPROP* values. Thus, *TPROP* was used to determine grids that have the potential to increase the percentage cover of the different types. Demand for grid cells with cultivated land and construction land cover was considerably higher than the actual cover in 2007, which experienced an increase in iteration. Some geophysical driving factors, namely elevation and slope, and some socioeconomic driving forces, namely population density and agricultural population, significantly contributed to the conversion.

### 3.4. AML Transformations Under Different Scenarios

**Figure 5:**
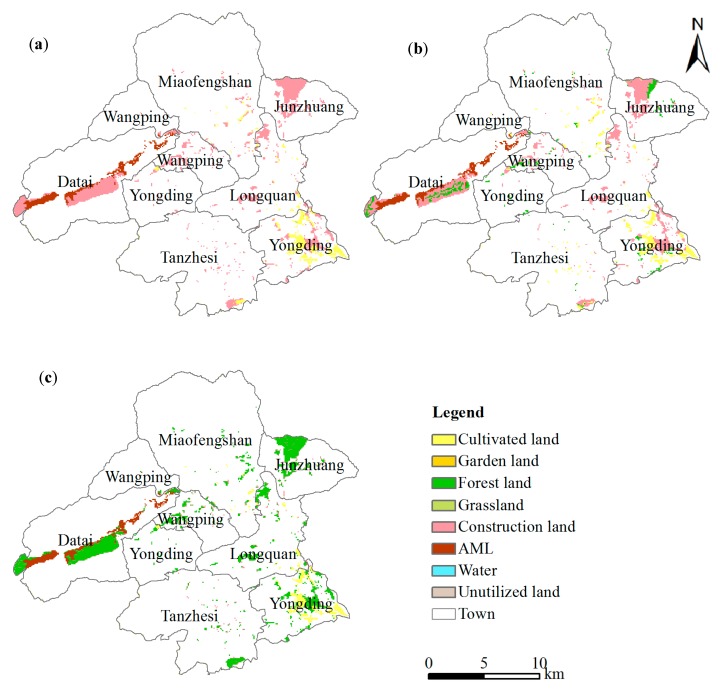
Spatial transformations of AML in 2007 under the three reclamation scenarios for 2020: (**a**) scenario 1; (**b**) scenario 2; and (**c**) scenario 3.

The spatial transformation of AML for 2020 under the three scenarios can be extracted by overlapping the layer of the original AML of 2007 with the simulated maps in Figure 4. The spatial transformations and areas of AML under the three reclamation scenarios for 2020 are shown in Figure 5 and Table 6, respectively.

#### 3.4.1. Scenario 1

The transformation of AML into cultivated land was seen in Yongding (Figure 5a). Owing to their flat terrain and transport accessibility, the conversion from AML into construction land mainly occurred in Junzhuang and Datai. In addition to cultivated land and construction land, the respective areas of reclaimed garden land, forest land, grassland, and water were 35 hm^2^, 152 hm^2^, 50 hm^2^, and 54 hm^2^. These areas were small and the spatial allocation of those types was not obvious.

#### 3.4.2. Scenario 2

AML was mainly reclaimed to cultivated land, forest land, and construction land (Figure 5b). Similar to scenario 1, the reclamation of cultivated land mainly occurred in Yongding, in the southeast region of the study area. In addition, some new reclaimed cultivated land was concentrated in the surroundings of the watershed in Miaofengshan owing to its good irrigation conditions. Forest land spread widely across Datai, which was suitable for reclamation to forest land and construction land.

#### 3.4.3. Scenario 3

AML was mainly reclaimed to cultivated land and forest land (Figure 5c). As for new cultivated land, similar conditions were observed in Yongding. Almost no spatial distribution of cultivated land was found in the other towns. Compared with the other two scenarios, demand for forest land was higher (Table 5). Assuming forest development followed the trend shown in the non-spatial module, some AML can be converted into forest land. By comparing the simulated scenario for 2020 with the actual map of 2007, we see that the conversion of forest land from the other types primarily occured in Yongding, Junzhuang, Miaofengshan, and Longquan.

### 3.5. Analysis of the Landscape Patterns under Different Scenarios

Nine landscape indices were selected to describe the characteristics of the landscapes and their components (Table 7). The spatial statistics quantify the differences between these simulated maps.

As shown in Table 7, scenario 3 had the largest average patch area and lowest size variability, whereas scenario 2 had a smaller average patch area but higher relative size variability. Considering the landscape shape index, scenario 2 was the highest because the landscape shape in this scenario was the most irregular and the length of its edges was the longest of the three simulated maps. The contiguity index of scenario 3 was higher than that of the alternatives. The contagion index ranked from highest to lowest in the order of scenario 3, scenario 2, and scenario 1. Moreover, scenario 3 had a high value for mean Euclidean nearest neighbor distance. The connectance index was computed as the proportion of functional joinings among all patches, where each pair of patches was either connected or not based on some criterion. The ranking of the connectance index for the three scenarios was the same as the mean Euclidean nearest neighbor distance. As for Shannon’s diversity index, it ranked from highest to lowest in the order of scenario 1, scenario 2, and scenario 3, which was the same for Shannon’s evenness index. These rankings suggested that scenario 1 had the most classes of units, with most of the units reporting a similar proportional area (evenness). Considering the selected landscape indices, scenario 3 is thus more suitable that its alternatives.

## 4. Discussion

### 4.1. Simulation Accuracy of the Combined Model

After getting the simulated maps through the modeling approach showed above, we examined the logistic regression results by using *ROC* curves; the *ROCs* for the eight land-use types were all above 0.7. Further, the accuracy of simulating whole types can be evaluated by using *Kappa* index [51]. The simulated map for 2013 was compared with the actual map using ENVI 4.8. The *Kappa* index was 0.90, which suggested that the model effectively captured future trends.

However, according to the MLS results, AML can only be shifted into four land-use categories, namely cultivated land, garden land, forest land, and construction land. As presented in Table 6, other land-use types such as grassland, water, and unutilized land showed small percentages, and their spatial allocations were not obvious. This may be caused by the relative error in the model (Table 5 and Table 6). We simulated changes in all land-use types by using the CLUE-S model, rather than only simulating AML shifts when running the model. To meet the requirements in the non-spatial model for the whole area, there was a balance between demand and the *TPROP* values of all the categories. We tried to overcome this shortcoming by only simulating the dynamics of AML without other land-use types. However, if we put the unique land-use type (*i.e.*, AML) into the model, the model cannot carry out the conversions between different land-use types without inputting various land-use types. Moreover, it cannot generate new land-use types during the simulation. The land-use types in the model must be consistent before and after a model run. Therefore, further research on parameter settings should be conducted to improve simulation accuracy.

### 4.2. Mechanisms of Landscape Pattern Changes under Different Scenarios

In the three scenarios, the reclamation of cultivated land mainly occurred in Yongding, located in the southeast region of the study area. The possible reason is that some geophysical driving forces, namely lower elevation and slope, and some socioeconomic driving forces, namely higher population density and agricultural population, significantly contributed to the conversion. In scenario 2, some new reclaimed cultivated land was concentrated in the surroundings of the watershed in Miaofengshan due to its good irrigation conditions. In addition, because of their flat terrain and transport accessibility, the conversion from AML to construction land mainly observed in Junzhuang and Datai. Further, reclaimed forest land was concentrated in Datai. A large amount of forest land spread widely across Datai because of the high connectivity of patches of the same type. Compared with the other two scenarios, demand for forest land in scenario 3 was higher (Table 5) and the conversion into forest land from other types mainly occurred in Yongding, Junzhuang, Miaofengshan, and Longquan, which have high transport accessibility.

Scenario 3 showed an advantage for most of the selected landscape-scale metrics. Mean patch size and patch size coefficient of variation provided simple statistics for the overall differences between the terrenes. Average patch area in scenario 3 was the largest and size variability was the lowest. The low number of patches in scenario 3 resulted in high mean patch size and low patch size coefficient of variation. The area-weighted mean patch contiguity index assessed the spatial connectedness, or contiguity, of the cells within the grid cell patch to provide an index of patch boundary configuration and thus patch shape. The contiguity index equaled 0 for a one-pixel patch and increases to a limit of 1 as patch contiguity, or connectedness, increases. Large contiguous patches resulted in larger contiguity index values; therefore, the contiguity index of scenario 3 was higher than those of other scenarios.

The contagion index has been widely used in landscape ecology given its effective summary of overall clumpiness on categorical maps. Contagion measures the extent to which patch types are aggregated or clumped (*i.e.*, dispersion); higher values of contagion may result from landscapes with a few large, contiguous patches, whereas lower values generally characterize landscapes with many small and dispersed patches. The higher value in scenario 3 may thus be attributed to the large patches of forest land caused by reclamation. The mean Euclidean nearest neighbor distance was perhaps the simplest measure of patch isolation and this equals the distance to the nearest neighboring patch of the same type according to the shortest edge-to-edge distance. The widespread forest land in scenario 3 significantly contributed to the high value of this index. The connectance index was calculated by using a threshold distance specified by the user and reported as a percentage of the maximum possible connectance, given the number of patches. The threshold distance was based on the mean Euclidean distance; thus, the ranking of the connectance index for the three scenarios was the same as the mean Euclidean nearest neighbor distance. As discussed above, scenario 3 dominated in most of the selected indices.

### 4.3. Implications for AML Management

From the aspect of landscape patterns, we advise that future ecological restoration policy in the area of AML transformation should concentrate on shifting AML to more forest land because of the closely combined and lowest variability patches as discussed above in order to maintain ecosystem stability and biological diversity [52]. As shown in Figure 2, forest land dominated in the study area, and therefore increasing the number of patches of the same type can enhance connectivity in the local area [53,54]. In China, one rule for the reclamation of AML is that reclaimed patches should be line with the surrounding land-use types, according to the *Management Approach to the Reclamation and Utilization of Abandoned Mine Land*. It is obvious that forest patches comprise a high percentage in the study area, and thus converting AML patches into forest would be consistent with the policy.

Moreover, in China, the concept of major function-oriented zones was proposed to achieve coordinated regional development and environmental protection based on territorial functions [55]. In 2012, the Beijing Municipal Government realized four such function-oriented zones in Beijing: the capital area, the urban extended zone, the new zone of urban development, and the ecological preservation zone. The Mentougou District was included in the latter. Transforming AML into more forest land has thus been identified as helping protect regional ecological safety and enhance ESV.

Further research is needed to consider a range of scenarios for AML reclamation and fully assess ecological risk under different scenarios [17] to formulate effective environmental policy. Further, we should further study the simulation accuracy, ecological parameterization, and ecological realism of land-use models. Moreover, the method of simulating AML conversions used in this study was based on incorporating the LP and CLUE-S models to obtain land requirements and spatial distributions, respectively. Future research that combines different land-use models and ecological models with more complex methods of simulating and assessing land-use scenarios [56] may provide a more comprehensive assessment of AML transformations.

## 5. Conclusions

To understand the potential impact of AML transformations under different land reclamation scenarios, we integrated the LP and CLUE-S models to provide a new approach for simulating AML shifts. Based on the results, nine landscape-scale metrics were then used to characterize the simulated maps. We found that the *ROC* and *Kappa* indices provided a good explanation of the conversion of land-use types under actual biophysical and socioeconomic driving forces. Therefore, the application in the case study demonstrates that the results of spatial transformations of land-use types under these three scenarios can be reliable.

Owing to the different characteristics of the three scenarios, however, AML transformations differed. New cultivated land dominated in scenario 1, while construction land and forest land accounted for major percentages in scenarios 2 and 3, respectively. From the analysis of the nine landscape indices, we can see that scenario 3 had an advantage in most with the patches combining most closely and with the lowest complexity.

From the perspective of landscape patterns, further ecological policy should focus on transforming AML into forest land to be in line with the surroundings in the study area. In China, a large number of mines have been or will be closed, thereby resulting in more AML. Therefore, it is urgent to formulate effective policies to restore AML in order to protect regional ecological safety and to keep people away from the soil pollution, soil erosion, and land desertification caused by abandoned land.

However, this study only identified the most suitable scenario from the perspective of land-use pattern analysis. Other factors such as reclamation investment costs and local policies should also be considered in future studies to explore the optimal land reclamation scenarios.

## Figures and Tables

**Table 1:** The main limiting factors of land reclamation and evaluation criteria of cultivated land, garden land, forest land, and construction land.

Limiting Factors	Index Grading	Evaluation for Cultivated Land	Evaluation for Garden Land	Evaluation for Forest Land	Evaluation for Construction Land
Slope	<5°	1	1	1	1
	5°–10°	2	2	2	2
	10°–15°	3	2	2	3
	>15°	N ^1^	3	3	N
Surface material composition	Loam, sandy loam	1	1	1	1
	Mixture of rock and soil	N	2 or 3	2 or 3	2
	Sand, gravelly soil	N	3	3	3
Soil organic matter	>1%	1 or 2	1	1	1
	0.5%–1%	3	2 or 3	2 or 3	2
	<0.5%	N	3	3	3
Soil layer thickness	>80 cm	1	1	1	1
	60–80 cm	1 or 2	2	2	2
	40–60 cm	2 or 3	2 or 3	2 or 3	3
	20–40 cm	3 or N	3 or N	3 or N	3 or N
	<10 cm	N	N	N	N
Irrigation and drainage condition	Fully satisfied	1	1	1	1
	Basically satisfied	2	2	2	2
	Without irrigation	N	3 or N	3 or N	N
Transport accessibility (distance to nearest road)	0–2000 m	1	1	1	1
	2000–4000 m	2	2	2	2
	4000–6000 m	3	3	3	3
	>6000 m	N	3 or N	3 or N	N
Land damage conditions ^2^	Light damage	2	2	2	1
	Moderate damage	3	3	3	2
	Severe damage	N	3 or N	3 or N	3

**^1^** N means it is not suitable for reclamation for the land-use category; **^2^** According to the Regulation on *Compiling Land Reclamation Plan in China* (TD/T 1031.1–2011), light damage refers to horizontal deformation of ≤8 mm/m, additional tilt (caused by mining activities) of ≤20 mm/m, subsidence of ≤2 m, or a decrease in production of ≤20%. Moderate damage refers to the horizontal deformation of 8–20 mm/m, additional tilt (caused by mining exploring activities) of 20–50 mm/m, subsidence of 2–6 m, or a decrease in production by 20%–60%. Severe damage refers to horizontal deformation of >20 mm/m, additional tilt (caused by mining exploring activities) of >50 mm/m, subsidence >6 m, or a decrease in production by >60%.

**Table 2:** Natural, economic, social, and total niches for the eight land-use types (CNY·hm^−2^) [42].

Land-Use Types	Natural Niche	Economic Niche	Social Niche	Total Niche
Cultivated land	16,453	6250	10,000	11,081.2
Garden land	17,082	8630	0	10,284.8
Forest land	30,011	430	1650	12,506.4
Grassland	19,110	330	1650	8106.0
Construction land	0	1000	58,594	12,118.8
AML	0	0	0	0
Water	12,803	68,880	1600	32,993.2
Unutilized land	11,906	0	0	4762.4

**Table 3:** ESVs for the eight land-use types (CNY·hm^−2^) [43].

	Cultivated Land	Garden Land ^1^	Forest Land	Grassland	Construction Land	AML	Water	Unutilized Land
ESV	3296.98	7516.28	11,735.57	4870.35	0	0	18,926.32	580.10

**^1^** Since Xie did not assign an ESV to garden land, the median ESV of cultivated land and forest land was taken to roughly estimate that of garden land in the study area.

**Table 4:** Landscape-scale graph metrics used in the study and their ecological significance.

Graph Metric	Ecological Description	Reference
Mean patch size	The area occupied by a particular patch type divided by the number of patches of that type.	[44]
Patch size coefficient of variation	Patch size standard deviation divided by the mean patch size; a measure of relative variability.	[44]
Landscape shape index	Landscape shape index provides a standardized measure of total edge or edge density that adjusts for the size of the landscape.	[44]
Area-weighted mean patch contiguity index	The contiguity index assesses the spatial connectedness, or contiguity, of cells within a grid cell patch to provide an index of patch boundary configuration and thus patch shape.	[45]
Contagion index	A quantitative index for measuring the degree of the clumpiness of the overall landscape patterns.	[46]
Mean Euclidean nearest neighbor distance	A patch-level distance (m) to the nearest neighboring patch of the same type, based on the shortest edge-to-edge distance, is averaged over all patches in the landscape.	[47]
Connectance index	Connectance is reported as a percentage of the maximum possible connectance given the number of patches.	[48]
Shannon’s diversity index	A measure of patch diversity in a landscape determined by both the number of patch types and the proportional distribution of the area among these types.	[49]
Shannon’s evenness index	A measure of patch distribution and abundance, which is equal to zero when the observed patch distribution is low and approaches one when the distribution of patch types becomes more even.	[50]

**Table 5:** The precision of the CLUE-S model simulation and its validation results in the non-spatial module.

Land-Use Types	Demand (hm^2^)			Simulation Results (hm^2^)			Relative Error (%)		
	Scenario			Scenario			Scenario		
	1	2	3	1	2	3	1	2	3
**Cultivated Land**	1656	1656	1656	1670	1675	1669	0.85	1.15	0.79
**Garden Land**	1678	1678	1678	1681	1671	1682	0.18	−0.42	0.24
**Forest Land**	29,738	31,035	32,784	29,805	31,161	32,802	0.23	0.41	0.05
**Grassland**	2643	1692	1109	2659	1702	1098	0.61	0.59	−0.99
**Construction Land**	6688	6689	6688	6689	6661	6680	0.01	−0.42	−0.12
**AML**	501	501	501	498	481	516	−0.60	−3.99	2.99
**Water**	955	1691	1109	950	1640	1080	−0.52	−3.02	−2.61
**Unutilized Land**	2775	1692	1109	2682	1643	1107	−3.35	−2.90	−0.18

**Table 6:** Areas of transformations of AML in 2007 under the three reclamation scenarios for 2020 (hm^2^).

Scenarios	Cultivated Land	Garden Land	Forest Land	Grass Land	Construction Land	AML	Water	Unutilized Land
Scenario 1	563	35	152	50	2246	440	54	33
Scenario 2	561	35	558	26	1879	424	67	23
Scenario 3	502	36	2484	10	10	458	55	18

**Table 7:** Landscape-scale graph metrics of the simulated maps.

Graph Metric	Scenario 1	Scenario 2	Scenario 3
Mean patch size	41.384	39.561	44.582
Patch size coefficient of variation	2077.569	2103.256	1989.408
Landscape shape index	18.200	19.381	17.554
Area-weighted mean patch contiguity index	0.827	0.816	0.834
Contagion index	54.968	56.091	60.348
Mean Euclidean nearest neighbor distance	373.062	360.209	377.284
Connectance index	1.193	1.183	1.250
Shannon’s diversity index	1.262	1.192	1.081
Shannon’s evenness index	0.607	0.573	0.520
